# Regulatory T Cell Dysfunction in Autoimmune Diseases

**DOI:** 10.3390/ijms25137171

**Published:** 2024-06-29

**Authors:** Dionne Y. Honing, Rosalie M. Luiten, Tiago R. Matos

**Affiliations:** 1 Department of Dermatology, Netherlands Institute for Pigment Disorders, Amsterdam University Medical Centers, University of Amsterdam, 1105 AZ Amsterdam, The Netherlands; d.y.honing@student.vu.nl (D.Y.H.); r.m.luiten@amsterdamumc.nl (R.M.L.); 2 Cancer Center Amsterdam, Amsterdam Institute for Infection and Immunity, 1081 HV Amsterdam, The Netherlands; 3 Sanofi, 1105 BP Amsterdam, The Netherlands

**Keywords:** regulatory T cell, autoimmune disease, immunology

## Abstract

Regulatory T cells (Tregs), a suppressive subpopulation of T cells, are potent mediators of peripheral tolerance, responsible for immune homeostasis. Many autoimmune diseases exhibit disruptions in Treg function or quantity, resulting in an imbalance between protective and pathogenic immune cells. Selective expansion or manipulation of Tregs is a promising therapeutic approach for autoimmune diseases. However, the extensive diversity of Treg subpopulations and the multiple approaches used for Treg identification leads to high complexity, making it difficult to develop a successful treatment capable of modulating Tregs. In this review, we describe the suppressive mechanisms, subpopulations, classification, and identification methodology for Tregs, and their role in the pathogenesis of autoimmune diseases.

## 1. Introduction

Regulatory T cells (Tregs) are a small yet critical subset of CD4^+^ T cells that possess immunosuppressive properties indispensable for the maintenance of immune homeostasis and self-tolerance. They are classically characterized by the expression of CD4, CD25, and FoxP3. CD4^+^CD25^+^FoxP3^+^ Tregs inhibit (1) excessive activation or proliferation of CD4^+^ helper T (Th) cells and cytotoxic CD8^+^ T cells, and (2) prevent B cell activation. The central role of Tregs in maintaining self-tolerance was initially made evident through animal models of autoimmunity. Deletion of Tregs by thymectomy in 3-day-old mice and adult rats resulted in spontaneous multiorgan autoimmune diseases that could be prevented by the adoptive transfer of CD4^+^ T cells [1]. This indicates that the thymus generates T cells with an autoimmune-protective function and that their development in mice can be disrupted by neonatal thymectomy.

Tregs can balance the immune response to foreign antigens and self-antigens, preventing excessive inflammation, which would otherwise lead to tissue damage or death. The potential loss of FoxP3 protein results in the lack of Tregs, which has fatal effects [2]. This was firstly observed in scurfy mice and subsequently in patients with immunodysregulation polyendocrinopathy enteropathy X-linked syndrome (IPEX syndrome). The genetic inactivating mutation of FoxP3 in IPEX syndrome causes spontaneous severe autoimmune diseases and allergies. In untreated scurfy mice, FoxP3 mutation leads to a defect in Treg function with exaggerated response of the effector T cells (Teffs), causing skin lesions, hair loss, growth retardation, lymphoproliferation, immunological abnormalities (e.g., Teff activation and the production of auto-antibodies), and death in 4–5 weeks [3,4]. Apart from FoxP3, several other molecules within Tregs are responsible for their function, including interleukin (IL)-2 receptor alpha chain (IL-2RA, CD25) and cytotoxic T-lymphocyte-associated protein 4 (CTLA-4). Similarly, fatal autoimmune diseases develop in individuals with mutation of CD25, whereas individuals with heterologous mutations of CTLA-4, including CTLA-4 haploinsufficiency, may develop autoimmune diseases of different spectrums [5,6,7].

The involvement of Tregs in the maintenance of self-tolerance is an essential part of the immune system’s regulatory network. Selective expansion or manipulation of Tregs is a promising approach in the search for new clinical applications for autoimmune diseases and graft versus host disease (GVHD). In this review, we discuss the suppressive mechanisms, subsets, classification, and identification of Tregs. Special attention is paid to the role of Tregs in the pathogenesis of autoimmune diseases, including type 1 diabetes (T1D), systemic lupus erythematosus (SLE), rheumatoid arthritis (RA), Crohn’s disease (CD), and ulcerative colitis (UC).

## 2. Suppressive Function of Regulatory T Cells

**Figure 1:**
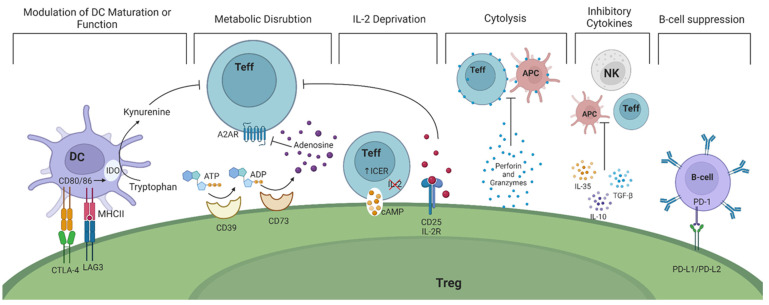
**Suppressive function of regulatory T cells (Tregs)**. Tregs can exert suppression on various cell types through both direct and indirect mechanisms. Tregs have been observed to have a direct effect on dendritic cells (DCs) via both cytotoxic T lymphocyte antigen-4 (CTLA-4) and lymphocyte-activation gene 3 (LAG-3). CTLA-4 and LAG-3 can interact with cluster differentiation 80/86 (CD80/86) costimulatory molecules and major histocompatibility complex (MHC) class II on DCs, respectively. As a result, indoleamine 2,3-dioxygenase (IDO) is generated, which in turn breaks down the essential amino acid tryptophan into kynurenine and inhibits the function and maturation of DCs as they become unable to activate T effector cells (Teffs). Treg cells can interfere with metabolic functions through the expression of ectoenzymes CD39/73, facilitating the generation adenosine. This immunoregulatory purine can then bind to adenosine receptor 2A (A2AR) present on Teff cells and reduce their proliferation. By high expression of CD25, Tregs can sequester interleukin (IL)-2 from the microenvironment, reducing Teff proliferation. Tregs have also been found to express elevated intracellular cyclic adenosine monophosphate (cAMP) levels. Through gap junctions they transfer this to Teffs, which leads to the upregulation of inducible cAMP early repressor (ICER), leading to inhibition of IL-2 transcription and consequently apoptosis due to IL-2 deprivation (indicated by red ‘X’). The release of perforin and granzymes causes damage to the target cell membrane, ultimately inducing apoptosis. Additionally, the secretion of anti-inflammatory cytokines, including IL-10, IL-35, and transforming growth factor-β (TGF-β), restrains the immune responses of T helper (Th)1 and Th17 cells and the production of interferon γ (IFN-γ) and IL-17, respectively. Lastly, Tregs have also been found to directly impact B cells through the interaction of programmed death ligand 1 (PD-L1)/programmed cell death protein 1 (PD-1). Abbreviations: ADP: adenosine diphosphate; APC: antigen presenting cell; AMP: adenosine monophosphate; ATP: adenosine triphosphate; A2AR: adenosine receptor 2A; cAMP: cyclic adenosine monophosphate; CD: cluster differentiation; CTLA-4: cytotoxic T lymphocyte antigen-4; DC: dendritic cell; ICER: inducible cAMP early repressor; IDO: indoleamine 2,3-dioxygenase; IL: interleukin; IFN: interferon; LAG3: lymphocyte-activation gene 3; MHC: major histocompatibility complex; NK: natural killer cells; PD-1: programmed death protein 1; PD-L: programmed cell death ligand; Teff: effector T cell; TGF-β: transforming growth factor-β; Treg: regulatory T cell.

The suppressive function of Tregs on Teffs, B cells, or antigen-presenting cells (APCs) cannot be attributed to a single effector molecule. Rather, Tregs exhibit a nuanced array of mechanisms that act on various immune and non-immune target cells and in different contexts. Figure 1 illustrates six widely acknowledged suppressive mechanisms employed by Tregs [8,9].

(1) Modulation of dendritic cells (DCs) in a cell—cell dependent manner ultimately prevents their maturation. This is accomplished through the expression of CTLA-4 and lymphocyte activation gene 3 (LAG-3) on the surface of Tregs. CTLA-4 and LAG-3 can interact with CD80/86 co-stimulatory molecules and major histocompatibility complex (MHC) class II on DCs, respectively [10,11]. As a result, the function and maturation of DCs are disrupted and they become unable to perform their effector function, namely activating Teffs. Studies in mice have shown that CTLA-4 is essential for the suppressive function of FoxP3^+^ Tregs while LAG-3 is required for maximal Treg activity in vitro and in vivo [12,13]. In humans, it is intriguing that only high levels of CTLA-4 expression are observed in CD45RO^+^FoxP3^high^ effector Tregs (Table 1) [14]. Recent evidence suggest that Tregs may exert a dual suppressive action via trogocytosis of APC membranes by depleting CD80/86 (in a CTLA-4 dependent manner) and stable peptide-MHCII complexes to simultaneously deprive co-stimulation and antigenic stimulation of both naïve and activated (antigen-specific) Tconvs [15,16]. Whether the interaction of APCs with CTLA-4 is indispensable for the in vitro suppressive function of Tregs and whether TCR/MHC peptide engagement is necessary or enhances Treg suppressive function remain to be elucidated.

(2) Tregs exert their suppressive function by disrupting metabolic pathways via the highly expressed ectoenzymes CD39/CD73 on their cell surface. These enzymes catalyze the hydrolysis of extracellular adenosine triphosphate (ATP) and adenosine diphosphate (ADP) to adenosine monophosphate (AMP) and subsequently adenosine, which binds to adenosine receptor 2A (A2AR), expressed on Teffs. The adenosine-A2AR signaling pathway ultimately suppresses Teff responses [36]. The expression of CD39 and CD73 have increasingly been used as markers of Tregs due to their potential contribution to the suppressive activity of Tregs (Table 2) [37,38].

It has also been postulated that reverse signaling mediated by CTLA-4-CD80/86 interaction may result in depletion of tryptophan inducing cell-cycle arrest, inactivity or anergy, and/or apoptosis of T-cells [53,54].
Upon activation, resting T cells rapidly upregulate their metabolic and biosynthetic machinery, including increased translational activity, prior to impending cell division. Recent data suggest that through mTORc signaling mediated by IL-10 and TGFβ, Tregs inhibit protein synthesis in effector T cells by preventing translation of mRNAs encoding components of protein synthesis machinery, thereby preventing proliferation of Teff cells before it has begun [55]. In addition, a functional mitochondrial complex-III in Tregs is needed to maintain immune regulatory gene expression and suppressive function of Tregs [56].

(3) Tregs inhibit Teffs through the deprivation of interleukin-2 (IL-2), a cytokine that plays a critical role in T cell activation and proliferation. Tregs upregulate IL-2 receptor (CD25), allowing them to bind IL-2 and prevent neighboring cells from receiving pro-inflammatory cytokine signals [57]. IL-2 is also necessary for the suppressive activity of Tregs since it activates and phosphorylates the signal transducer and activator of transcription 5 (STAT5), leading to FoxP3 gene transcription [58]. Signaling through IL-2 receptors significantly upregulates CD25 expression, enhancing Tregs’ ability to bind IL-2 and further deprive surrounding Teffs [59,60]. Mice with deficiencies in either IL-2 or its receptor display enlarged peripheral lymphoid organs, impaired activation-induced cell death, and autoimmune disorders, all attributed to a diminished Treg generation [5].
(4) Tregs also secrete various anti-inflammatory cytokines, including IL-10, IL-35, and transforming growth factor-β (TGF-β). These cytokines restrain the immune responses of T helper (Th)1 and Th17 cells and the production of interferon γ (IFN-γ) and IL-17, respectively [19].
(5) The release of granzyme A, granzyme B, and perforin by Tregs directly kills Teffs and B cells by the induction of cytolysis [19].

Extracellular vesicles (EVs) derived from Tregs is another mode deployed by Tregs to modulate immune responses. Treg-derived EV cargo includes immunomodulatory proteins and cytokines such as CD25 and IL-35, and miRNAs that reduce T-cell proliferation, IL-2 and IFNγ release, modify DC function, and promote IL-10 production by murine DCs [53,61,62].

(6) Tregs have also been found to directly impact B cells through the interaction of programmed death-ligand 1 (PD-L1)/programmed cell death protein (PD-1) [63]. Tregs indirectly prevent B cell activation by inhibiting T follicular helper (Tfh) and Th-cells. This results in the inability of Tfh cells to activate B cells and Th cells to produce antibodies [63,64].

The timing of their egress from thymus and proper and timely access to peripheral tissues is also essential for Treg cells to establish and maintain immune homeostasis. A paucity in colonization by early wave of thymic Tregs that are enriched for reactivity against AIRE-dependent antigens, for example, can lead to development autoimmune-mediated target tissue injury [65,66]. Similarly, accumulation of Tregs during the neonatal period is essential in maintaining tolerance [65,67]. In addition, the high and constitutive expression of effector molecules needed for Treg suppression (such as CD25, CTLA-4, IL-10) give Tregs a head start in suppressing T-cell activation, which acquire these suppressive molecules much later, during their activation [65].

In addition to their canonical roles in immune regulation, which indirectly enhance tissue resilience during inflammation, Tregs are increasingly recognized for their direct contributions to tissue maintenance and repair across various organs. Tregs within white adipose tissue express the transcription factor peroxisome proliferator-activated receptor gamma (PPARγ) and play a role in regulating metabolic functions, partly by producing IL-10 [68]. Those residing in the bone marrow help maintain hematopoietic stem cell quiescence by enzymatically converting extracellular ATP to adenosine through CD73/CD39 [69]. Tregs in injured muscle, brain, and lung facilitate tissue regeneration, partially via secretion of the growth factor amphiregulin [70,71]. Additionally, skin-resident Tregs employ diverse mechanisms, such as activating notch or TGF-β receptor signaling in hair follicle stem cells (HFSC) through ligand provision, to support the stem cell niche and promote hair follicle and epithelial barrier regrowth [72]. More recently, it has been suggested that murine skin Tregs immunologically protect the HFSC niche, conferred by the elevated expression of CD25 on Tregs [73].

## 3. Molecular Signatures for Identifying Regulatory T Cells

Tregs are a heterogeneous group of phenotypically distinct subsets capable of residing in both lymphoid and non-lymphoid organs. There are many ways to classify Tregs. Initially, in 2001, human Treg cells were characterized as CD4^+^CD25^+^ T cells, based on the discovery in 1995, that IL-2RA (CD25) is expressed constitutively on mouse Tregs [41,74,75]. Similarly, in 2003, FoxP3 was identified as a master control gene for development and function of Tregs in mice [2,76]. Since then, further investigation confirmed that FoxP3 expression is primarily restricted to CD4^+^CD25^+^ cells, serving as a specific marker for human Tregs [77].

The functional analysis of Tregs is hindered by FoxP3 being an intracellular protein that cannot be used as a marker for cell sorting in humans. Furthermore, human T cells expressing FoxP3 exhibit similarities to conventional T cells (Tconvs), since activated Tconvs have the potential to express low levels of FoxP3 [78].

Therefore, other methods or cell surface markers are crucial for identifying viable Tregs. Another method used to identify Tregs involves examining the epigenetic regulation of the FoxP3 locus. In Tregs, the FoxP3 gene promoter region is typically hypomethylated, which is associated with stable FoxP3 expression and Treg lineage stability. Therefore, assessing the demethylation status of the FoxP3 locus could be seen as an absolute marker for Foxp3^+^ Tregs [79]. In mice, CD25 is a pivotal marker for identifying and isolating CD4^+^FoxP3^+^ Tregs from other immune cell populations (Table 2) [41]. However, the use of CD25 expression as a marker is limited in human studies as up to 30% of human peripheral blood CD4^+^ T cells express CD25, and many of these cells appeared to be effector and memory cells [17,42]. These CD25 expressing T cells can be further classified into CD4^+^CD25^high^ and CD4^+^CD25^low^. The CD4^+^CD25^high^ T cell subset comprises approximately 1–2% of the total circulating CD4^+^CD25^+^ T cells, and only these cells have been shown to exhibit strong in vitro regulatory function in humans and can therefore be considered as Tregs [17,42]. No clear definition for the boundary between CD25^+^ and CD25^high^ expression has yet been achieved, making it difficult to analyze the number and function of Tregs, especially in inflamed conditions where activated T cells too express CD25 [42].

In experimental setups, Tregs are commonly identified by the dual expression of FoxP3 and CD25. However, studies have highlighted discrepancies in CD25 and FoxP3 expression within CD4^+^ T cells of humans and mice, suggesting a lack of correlation [43,76]. To refine the characterization of human Tregs, recent research has emphasized the importance of low levels of CD127 (IL-7 receptor α-chain) combined with high CD25 expression to isolate FoxP3 expressing CD4^+^ T cells effectively (Table 2) [43,44]. Utilizing CD25^+^ and CD127^−^ as surface markers for Treg isolation offers practical advantages, obviating the need for additional intracellular staining of FoxP3, thus maintaining cell viability for downstream analyses. Nevertheless, it has also been suggested that the label CD4^+^CD25^+^CD127^low^ T cells may lack specificity, as they encompass non-regulatory FoxP3^+^CD4^+^ T cells capable of producing pro-inflammatory cytokines (IL-17, high amounts of IL-2 and IFN-γ) and failing to suppress Teffs in vitro [14]. Further complexity arises from the observation that FoxP3 is not expressed in 34% of CD127^−^ T cells, as CD4^+^ Tconvs often downregulate CD127 post-activation [80]. Whether this is due to the vast heterogeneity within the FoxP3^+^CD25^high^CD127^low^ population of Tregs and/or technical inadequacies in sorting and identifying the correct population of T cells needs further evaluation.

Besides these commonly used markers, there are numerous T cell surface markers that can assist in distinguishing Tregs, such as CTLA-4 and glucocorticoid-induced tumor necrosis factor-related receptor (GITR), helios, neuropilin-1 (Nrp-1), tumor necrosis factor receptor superfamily member 4 (OX40), CD39, and CD73 [17]. CTLA-4 and GITR, which are known to be present on Tregs, are also expressed on potent Teffs, making it challenging to identify and determine the functional role of these cells. Helios is a transcription factor whose expression has been associated with thymic-derived (natural) Tregs, although its specificity for Tregs is debated [81]. Nrp-1 is a transmembrane protein that is highly expressed on Tregs and is thought to be involved in their development and function. CD39 and CD73, ectoenzymes expressed on Tregs, respectively, hydrolyze ATP, and catalyze the conversion of AMP to adenosine, contributing to their regulatory role [37].

While FoxP3 expression is generally considered a specific Treg marker since it correlates with suppressor activity independent of CD25 expression, assessing the demethylation status of FoxP3 locus can provide complementary information about Treg stability and function. However, it remains unclear what is the most accurate marker combination to identify these cells [40]. Matos et al. revealed that Treg subsets exhibit significant heterogeneity beyond that which standard flow cytometry characterization with a limited marker panel can detect. Additionally, this heterogeneity varies considerably among individuals [82]. Therefore, it is difficult to reach a single unanimous agreement on Treg labeling due to the high heterogenicity of Treg subsets and function, and various methodologies used, such as intracellular versus cellular membrane staining or immunohistochemistry.

## 4. Subsets of Regulatory T Cells

### 4.1. FoxP3^+^CD4^+^ Regulatory T Cells

While in mice FoxP3 expression is restricted to Tregs, in humans, FoxP3^+^ is not only expressed in Treg cells but can also be expressed at a low level among activated Tconvs [78]. Miyara et al. defined three distinct subpopulations of human FoxP3^+^CD4^+^ T cells with a precise phenotype and fate. The first two subsets, (1) CD25^++^CD45RA^+^FoxP3^low^ resting Tregs and (2) CD25^+++^CD45RA^−^FoxP3^high^ activated/memory Tregs, are both suppressive in vitro. The third group includes (3) non-suppressive cytokine secreting CD25^++^CD45RA^−^FoxP3^low^ T cells (Table 1) [14]. Naïve Tregs are CD4^+^ T cells that have not yet encountered their conjugate antigen and thus exhibit a relatively undifferentiated phenotype and lack suppressive activity. In humans, they express low levels of Treg-specific markers such as CD25, FoxP3, and CTLA-4 (Table 1) [17]. Furthermore, certain Tregs are classified as apoptotic Tregs, presenting high-levels of CD95 (also known as Fas) and low-levels of BCL-2. These apoptotic Tregs undergo programmed cell death, which is an important mechanism for maintaining immune homeostasis [18,83].

### 4.2. Subpopulations of CD4^+^ Regulatory T Cells

It is well established that Tregs can be broadly classified into thymically derived natural Treg (nTregs) and inducible Tregs (iTreg) cells. Both nTregs and iTregs are characterized by high expression of CD25, FoxP3, CTLA-4, and GITR. However, a combination of novel TCR transgenic mice with specific self-antigens and conventional mouse models might enable the discrimination of iTregs from nTregs by their reduced expression levels of PD-1, the transcription factor helios, and the surface antigen neuropilin-1 (Nrp1) (Table 1) [84]. 

However, studies argue against helios being a suitable marker for differentiating between nTregs and iTregs, as its expression can be induced during T cell activation and proliferation but diminishes under resting conditions. This phenomenon is not only observed in human and murine Tregs but also in CD4^+^ and CD8^+^ T cells [81]. Alternatively, Nrp1 expression on nTregs may offer a more reliable marker for identifying these cells. Still, conflicting data persist, demonstrating that iTregs produced under inflammatory conditions express varying levels of Nrp1 [27].

**Figure 2:**
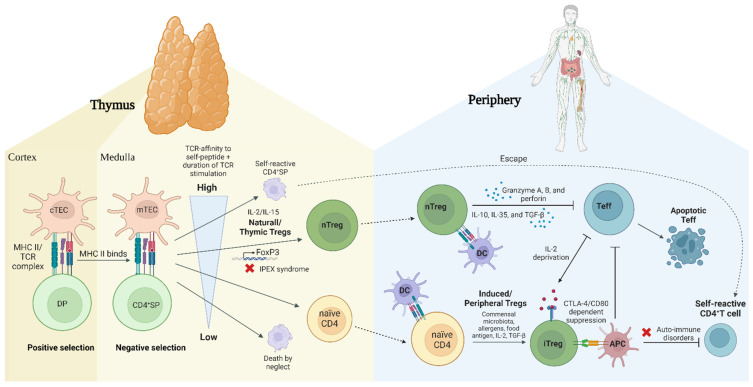
**Schematic diagram of Treg development**. During the rearrangement of the T cell receptor (TCR), thymocytes go through two stages known as positive and negative selection. During positive selection, double positive (DP) T cells receive a survival signal when they bind with sufficient affinity to cortical thymic epithelial cells (cTECs) expressing class I or class II MHC molecules along with self-peptides. Negative selection occurs in the medulla when the TCR of a thymocyte binds with high affinity to a peptide-MHC ligand on medullary thymic epithelial cells (mTECs), resulting in a self-reactive CD4+ single positive (SP) T cell and subsequent apoptotic cell death. As this process is not always effective, some self-reactive T cells evade elimination and enter the periphery, possibly causing autoimmune diseases. High-affinity tissue-restricted binding of MHC II/TCR and subsequently IL-2 or IL-15 signaling result in nTreg development by upregulation of FoxP3 and CD25. Low-affinity binding results in naïve CD4+ T cells. These naïve CD4+ T cells may develop in the periphery to iTregs in response to environmental factors such as commensal microbiota, allergens, food antigens, IL-2, and TGF-β. In the periphery, Tregs play a role in suppressing immune responses directed against both self and non-self-antigens. This involves the secretion of inhibitory cytokines (IL-10, IL-35, and transforming growth factor-β (TGF-β)), inhibition of effector cells through granzyme-dependent and IL-2 cytokine-deprivation mediated mechanisms, and modification of DC function and maturation through cell-contact-dependent interactions. Abbreviations: CD: cluster differentiation; cTEC: cortical thymic epithelial cells; DP: double positive (CD4+CD8+); IL: interleukin; iTreg: induced Treg; MHC: major histocompatibility complex; mTEC, medullary thymic epithelial cells; nTreg; natural Treg; SP: single positive (CD4+ or CD8+); TCR: T cell receptor; Teff: effector T cell; TGF-β: transforming growth factor-β; Treg: regulatory T cell.

**nTregs** mature in the thymus, whereas iTregs arise from naïve T cell precursors in peripheral tissues (Figure 2) [85]. The generation of nTregs depends on the presentation of a thymic self-peptide/MHC activation, prompting the differentiation of precursor cells into competent antigen-specific suppressive Tregs. In normal mice, this development acquires hypomethylation patterns in Treg function-associated key genes, such as CD25, CTLA-4, EOS, and helios, before FoxP3 expression [86,87]. The nTreg subpopulation in humans and mice can be further classified into ICOS^+^, ICOS^−^, HLA-G^+^, HLA-DR^+^, and HLA-DR^−^ subsets with different suppressive mechanisms (Table 1) [21,22,24]. In several autoimmune diseases, nTreg instability and plasticity may be due to epigenetic modifications and mutations of the FoxP3 gene (see Section 4).

**iTregs** arise from naïve T cell precursors in peripheral tissues (Figure 2). Human and mouse iTregs can be further classified into subpopulations of IL-10-secreting CD4^+^ regulatory 1 T cells (Tr1 cells), TGF-β-secreting regulatory T cells (Th3), and IL-35 secreting regulatory T cells (iTr35) (Table 1) [33]. While the differentiation of nTregs relies on interactions with self-peptide-MHC complexes, peripheral iTregs’ differentiation most likely occurs in response to non-self-antigens, such as commensal microbiota, food, and allergens (Figure 2). Under these conditions, human naïve CD4^+^ cells could induce the expression of both CD25 and FoxP3, obscuring the identification of FoxP3^+^ T cells as pure Tregs [88]. Additionally, the differentiation of iTregs is influenced by signaling strength through the abundance of antigens or the presence of IL-2, TGF-β, and IL-35 [33,89,90]. The phenotypic heterogeneity of iTregs enables them to sustain immune tolerance during extreme inflammatory conditions, and aids nTregs in the restoration of immune tolerance when required [91].

The origin of iTregs remains a topic of debate, with questions surrounding whether they emerge from a pre-committed cell lineage or if any T cell has the potential to convert into an iTreg depending on the cytokine milieu [92,93]. One hypothesis is that CD4^+^FoxP3^−^ Tconvs may have a Treg ‘epigenome’ imprinted on them during thymic development, making them more likely to differentiate into iTregs upon receiving appropriate TCR signals in the periphery [87]. Contrarily, studies have demonstrated that thymic pre-activation is not a prerequisite for iTreg conversion in mice. Rather, the majority of murine iTregs originate from T cells that have recently exited the thymus. The primary determinant for iTreg generation is their lower peptide-major histocompatibility complex (pMHC) affinity (Figure 1). Additionally, the antigen dose contributes, with lower doses generally promoting the formation of FoxP3^+^ iTregs [94,95].

**Treg-of-B cells:** B cells also play a role in the development and amplification of Tregs in both mice and humans. Naïve B cells in the spleen and Peyer’s patch act as APCs and can convert CD4^+^CD25^−^ T cells into CD4^+^CD25^+^FoxP3^−^ Tregs (Treg-of-B cells) through a cell—cell contact-dependent manner [35]. These Treg-of-B cells secrete IL-10, and express LAG-3, GITR, CTLA-4, inducible co-stimulators (ICOS and CD278), OX40, and PD-1. Preventative transfer of Treg-of-B cells in mice effectively prevented the development of Th2-mediated allergic asthma [96], Th1/Th17-mediated inflammatory bowel disease [97], and collagen-induced arthritis [98]. These findings demonstrate the ability of Treg-of-B cells to exert suppressive functions in both in vitro and in vivo.

**Tissue-resident Foxp3+ Tregs** (TR-Treg) have garnered considerable attention in the past decade, representing a resident population of antigen-experienced memory T cells situated within non-lymphoid tissues. Upon activation, Treg cells migrate into inflamed or infected target tissues, where they receive tissue-specific signals facilitating their further differentiation into TR-Treg cells. These signals are unique to each tissue and are believed to activate tissue-specific gene signatures in Treg cells [99]. These cells play a pivotal role in protecting the host against pathogens re-entering tissue sites, primarily skin, muscle, and visceral adipose tissue (VAT). Notably, they exhibit transcriptionally distinct programs compared to circulating memory counterparts, indicating unique mechanisms governing their homeostasis and functions. While numerous reports have described the presence of Tregs detected in tissues, the concept of TR-Treg cells was first elucidated in a study by Feuerer et al. The study investigated a distinct Treg cell population in VAT following the surprising observation that more than 50% of CD4 T cells in epididymal fat pads expressed FoxP3 [100]. These Treg cells exhibited comparable suppressive capacity but displayed distinct transcriptional profiles compared to Treg cells isolated from lymphoid tissues. It is believed that VAT Tregs originate from the thymus and express high levels of helios and neuropilin-1 (Nrp-1), markers typically associated with thymus-derived Treg cells. Future research will elucidate the mechanisms by which TR-Treg cells maintain residency within tissues, focusing, for example, on analyzing TCR repertoires and identifying tissue-specific antigens crucial for guiding the differentiation pathways of TR-Treg cells.

**Antigen-specificity of Tregs** adds an additional layer of complexity to understanding Tregs, especially pertinent in the context of autoimmune diseases and their treatment. Defects in Tregs may primarily affect antigen-specific subsets. For instance, in type 1 diabetes, islet antigen-specific Tregs have more potent therapeutic effects than polyclonal ones, and engineering Treg specificity for islet antigens using a T cell receptor-like CAR is a promising therapeutic approach for the prevention of autoimmune diabetes [101]. Investigating antigen-specific Treg populations holds promise for elucidating disease mechanisms and identifying potential therapeutic targets tailored to specific autoimmune antigens. While the majority of studies analyzing antigen-specific Tregs in relation to autoimmune diseases primarily focus on blood Tregs, there exists an entire field dedicated to investigating Tregs within tissues.

### 4.3. Plasticity of Treg Phenotypes

While the transcription factor FoxP3 was initially regarded as the primary regulator of CD4 Treg development and function, a more intricate regulatory network has now emerged. Rather than being governed by a singular factor, the Treg suppressive program is orchestrated by a combination of transcription factors, genetic and epigenetic elements, and signals from the tissue microenvironment. The complexity underlying the Treg suppressive phenotype suggests that loss of Treg lineage commitment can stem from either FoxP3 downregulation/deficiency or various alternative genetic and transcriptional dysregulations. However, the precise mechanisms underlying genetic and epigenetic variations that influence Treg development, lineage commitment, and function during normal homeostasis, and their impairment in autoimmune and inflammatory diseases, remain only partially understood.

While epigenetic and genetic mechanisms ensure continued FoxP3 expression in Tregs resulting in strong lineage stability, Tregs also have remarkable ability to adapt to their microenvironment phenotypically and functionally (for example, inflammatory milieu favoring Th cell development) to maintain their suppressive function. This plasticity of Treg phenotypes can be delineated into two categories: healthy and useful plasticity, seen in Th-like Tregs under healthy conditions and unstable plasticity, observed in ex-Tregs.

Useful plasticity is seen in environments with high levels of pro-inflammatory cytokines such as IL-6, IL-1β, and IL-12, where a subset of Tregs can modify their functionality from suppressive into pro-inflammatory effector capacity cells. For example, while maintaining FoxP3 expression, Tregs can acquire an effector Th-like phenotype and produce pro-inflammatory cytokines, such as IFN-γ (in Th1-like Tregs), IL-17 (in Th17-like Tregs), and IL-13 (in Th2-like Tregs) (Table 3) [102,103,104]. For example, within the gut, the delicate equilibrium between Treg and Th17 cells is essential for host defense and tolerance. The plasticity of these cells is influenced by factors like the cytokine milieu, metabolites (such as retinoic acid), microbial products, and the gut microbiota itself. The flexibility of Th17 cells to switch between pro-inflammatory and regulatory roles allows them to contribute to both immune defense against pathogens and the maintenance of intestinal homeostasis by regulating immune responses in the gut. Disturbances in this plasticity are implicated in the pathogenesis of IBD, where an imbalance between regulatory and effector T cells leads to chronic inflammation [105]. Indeed, in patients with IBD, a lower ratio of Treg/Th17 cells has been observed [106] and may aid in exacerbating intestinal inflammation.

On the other hand, unstable plasticity can lead to the loss of FoxP3 expression in Tregs, resulting in the acquisition Th cell-like phenotypes (ex-FoxP3 cells) (Table 3). This phenomenon is observed in both homeostatic and autoimmune conditions in unmanipulated mice, where these cells produce pro-inflammatory cytokines such as IL-2 and TNF (Table 3) [110]. The generation of these ex-FoxP3 cells is accelerated during T1D in mice. In T1D, ex-FoxP3 cells acquire an effector-memory phenotype, produce harmful cytokines, and may induce the development of autoimmunity. The exact molecular mechanisms underlying ex-FoxP3 generation are not yet fully understood, but it is conceivable that a deficiency of IL-2 signaling among Tregs within affected tissues could lead to a disruption of the positive feedback loop that governs FoxP3 stability [110,112]. A separate murine study has demonstrated that elevated levels of IL-6 relative to IL-2 can lead to the downregulation of FoxP3 expression in CD4^+^FoxP3^+^ T cells, resulting in their transition into ex-FoxP3 cells, although direct observations of such ex-FoxP3 cells in human autoimmune diseases are currently lacking [91]. If they do indeed exist, they are more likely to be localized within inflammatory tissues. In the context of Treg heterogeneity, IL-6 can also be essential for a certain subset of Tregs. A study by Becker et al. discovered that muscle-resting Tregs require IL6Rα signaling to control muscle function and regeneration in mice lacking IL6Rα on T cells (TKO) [113].

Moreover, Tregs exposed to prolonged or excessive inflammatory cytokines can become exhausted and lose their ability to regulate the immune response effectively (Table 3), potentially leading to a breakdown in immune tolerance and development of autoimmune diseases.

Studies on the characterization of mechanisms underlying Treg plasticity could provide new insights into the maintenance and restoration of self-tolerance. Many questions remain to be clarified. What are the molecular mechanisms underlying ex-FoxP3 cell generation? Do ex-FoxP3 Tregs play a role in the pathogenesis of autoimmune diseases? Is it possible to utilize Treg plasticity to develop new therapeutic strategies for the enhancement of Tregs to treat autoimmune diseases?

## 5. Regulatory T Cells Dysfunction in Autoimmune Diseases

### 5.1. Type 1 Diabetes

T1D is an autoimmune disease where inflammatory cells infiltrate pancreatic islets, resulting in the destruction of insulin-producing β-cells. One early report found that FoxP3^+^ Tregs (defined as CD4^+^CD25^+^ T cells) were reduced in individuals with T1D [114]. However, the use of more accurate markers to characterize these Tregs, including low CD127 expression and FoxP3 expression, has resulted in a consensus that the overall frequency of FoxP3^+^ Tregs remains unchanged in individuals with T1D [115,116,117,118]. FoxP3^+^ Tregs are not simply a homogeneous population of cells with a shared phenotype but are in fact a diverse mixture of cellular phenotypic subtypes that reflect different states of maturation, differentiation, and activation, or use different methods or targets of suppression [119]. Consequently, it is plausible that a change in the balance or frequency alteration of a Treg subtype could be observed in T1D. Indeed, findings by Okubo et al. showed a decrease in the frequency of activated FoxP3^+^ Tregs in peripheral blood of individuals with T1D compared to those without T1D [120]. Children with T1D also seem to have an increased proportion of IL-17-secreting CD45RA^−^CD25^int^FoxP3^low^ cells [88].

Circumstantial evidence suggests that Tregs are involved in the pathogenesis of T1D. Blocking the CD28-B7 pathway or inhibiting IL-2 activity in non-obese diabetic (NOD) mice has shown to accelerate the development of diabetes. This acceleration is attributed to the disruption of Treg development, Treg homeostasis, and an age-dependent decline in Treg function [86,87,121]. In addition, a progressive decrease in the Treg/Teff ratio is seen in inflamed pancreatic islets of Langerhans. Administration of low-dose IL-2 promoted Tregs survival and protected mice from developing diabetes. Further mice studies showed that low-dose IL-2 prevented the onset of T1D and preserved beta cell functions in NOD mice by increasing the numbers of Tregs in the pancreas and inducing expression of Treg-associated proteins (e.g., FoxP3, CD25, CTLA-4, ICOS, and GITR) in these cells. Transcriptome analyses have revealed that low-dose IL-2 has a significantly greater impact on gene expression in Tregs compared to Teffs, suggesting that nonspecific activation of pathogenic Teffs is unlikely [89,90]. In children with new-onset T1D, the administration of autologous CD4^+^CD25^high^CD127^−^ has been demonstrated to be safe and well-tolerated, while in some T1D patients’ insulin requirement significantly decreased after receiving Treg therapy suggesting reduced disease severity [91,96]. However, in a recent Phase 2 clinical trial in children and adolescents with recent-onset T1D, a single dose of CD4^+^CD25^hi^CD127^lo/−^ autologous expanded polyclonal Tregs did not prevent decline in β-cell function, despite the suppressive capacity of the expanded Tregs in vitro [122]. Together, these results suggest that Treg dysfunction, Treg frequency, and IL-2 production may contribute to the pathogenesis of T1D.

Low-dose IL-2 has demonstrated success in reverting the improper balance of Tregs/Teffs in NOD mice. Due to its short half-life (<15 min), it needs to be infused continuously or injected frequently to maintain its effect, which can result in undesired stimulation of non-Tregs. This has led to mutant forms and fusions of IL-2 with carrier proteins that possess a much longer half-life and greater selectivity for Tregs. A recently introduced fusion protein, mIL-2/CD25, could periodically dissociate into a biologically active monomer, allowing a long-lived IL-2R agonist that promotes preferential interaction with CD25-expressing cells, and thus facilitating a targeted response in Tregs in NOD mice [123]. mIL-2/CD25 greatly improved pharmacokinetics and pharmacodynamics when compared with recombinant IL-2 as it not only expanded Tregs but also increased their activation and migration into lymphoid tissues and the pancreas. This, in combination with elevated levels of IL-10 production by activated Tregs, might lower islet inflammation while simultaneously promoting an increase in Tregs within the inflamed islets. mIL-2/CD25 treatment may potentially decrease the risk of diabetes by dampening Tfh cell function, thereby reducing the production of anti-insulin autoantibodies in NOD mice. However, it is important to note that the role of autoantibodies as drivers of the T1D pathogenesis is highly debated [124]. Although monotherapy with mIL-2/CD25 has shown promise, only some NOD mice were fully responsive. Similar variability in Treg expansion and responsiveness may be present in humans, which highlights the need to identify biomarkers that can predict the response to mIL-2/CD25 as well as low-dose IL-2 treatment in T1D and other autoimmune diseases.

### 5.2. Systemic Lupus Erythematosus

SLE is a multi-system autoimmune disease that can affect various organs through the production of autoantibodies and deposition of immune complexes in tissues, leading to complement activation, accumulation of neutrophils and monocytes, and activation of self-reactive lymphocytes. Most studies have been described a decreased percentage of activated Tregs (CD25^+++^CD45RA^−^FoxP3^high^), an increased percentage of resting Tregs (CD25^++^CD45RA^+^FoxP3^low^), and a notable increase in the percentage of non-suppressive cytokine-secreting T cells (CD25^++^CD45RA^−^FoxP3^low^) in peripheral blood [125]. However, others have reported unaltered or even increased Treg numbers in SLE patients compared to healthy controls [126,127].

Lupus-prone mice ((NZBxNZW) F_1_ mouse model) showed a progressive deficiency in the number of Tregs in the lymph nodes and peripheral blood as they aged and the disease progressed, a progressive decline in IL-2-producing CD4^+^ T cells, while the suppressive function of Tregs remained similar [128]. Neutralization of IL-2 levels in healthy mice caused a strong and persistent reduction in the percentage of CD4^+^FoxP3^+^CD25^+^ Tregs in peripheral blood, lymph nodes, and later also in the spleen. This reduction in Tregs causes an imbalance of Tregs and Teffs, strongly accelerating disease progression. Conversely, the administration of IL-2 exhibited a partial restoration of the equilibrium between Tregs and Teffs. This was accomplished by homeostatic proliferation of endogenous Tregs, which effectively impeded the progression of the disease. In addition, adoptive transfer of IL-2 pre-activated Tregs delayed disease progression and significantly increased the survival time. This confirms that CD4^+^FoxP3^+^ Tregs derived from lupus-prone mice remained functionally intact and served as a physiologically relevant inhibitor of disease progression [128]. Nevertheless, the efficacy of adoptive transfer of Tregs in human SLE patients has been inconsistent, with conflicting reports, potentially due to differences in isolation and purification strategies applied for the enrichment of FoxP3^+^CD25^+^ Tregs prior to infusion.

### 5.3. Rheumatoid Arthritis

Similar to SLE patients, reports on Tregs numbers in patients with RA are quite controversial. RA is a systemic and heterogeneous chronic inflammatory condition characterized by the clinical manifestation of symmetrical polyarthritis, which can cause cartilage and bone damage as well as disability in patients. Some studies of patients with RA reported an increase, while others observed a decrease or no significant Treg changes in the synovia as well as in the peripheral blood [129,130,131,132]. Contradictory results were also reported for the functional characteristics of Tregs of RA patients [133,134]. The absence of a distinct reduction in Treg number or function in RA patients suggest that Treg dysfunction may be unique to the disease. Morita et al. performed a meta-analysis on the quantity and proportion of Tregs in individuals with RA, finding fewer Tregs in peripheral blood but an increased number in synovial fluid [135].

The controversial nature of these results can be attributed, firstly, to the inherent heterogeneity of the disease and to the absence of consensus on specific Treg markers, as previously discussed. Notably, in the human system, CD25 and FoxP3 expression can be transiently induced upon T cell activation, as highlighted earlier. Consequently, in autoimmune conditions like SLE, conclusions must be approached with caution due to the prevalent phenomenon of heightened T cell activation.

In the context of JIA, Tregs are characterized by decreased FoxP3 stability and CD25 expression [136], altered cytokine and chemokine production [137], and decreased responsiveness to IL-2 [136], indicating impaired Treg function. However, it is reported that Tregs derived from JIA synovial fluid and peripheral blood are suppressive outside the joint, when in vitro [137,138]. This suggests that the inflammatory microenvironment is most probably responsible for the improper function of Tregs in joints of JIA and RA patients [39]. Komatsu et al. showed that under arthritic conditions mediated by IL-6, CD4^+^CD25^low^FoxP3^+^ T cells lose FoxP3 expression (ex-FoxP3 cells), undergo differentiation into Th17 cells, and accumulate in inflamed joints [33,91]. Remarkably, adoptive Treg transfer not only suppressed T and B cells but also directly reduced osteoclast-mediated bone destruction, protecting the joints from injury in a mouse model of collagen-induced arthritis (CIA) [139]. Despite the high levels of IL-6 found in inflamed joints, adoptive Treg transfer in a murine model of CIA showed inhibitory effects on arthritis and effectively prevented the development of CIA. This indicates that systemic autoimmune diseases associated with elevated levels of pro-inflammatory cytokines may be ameliorated by Tregs [139].

### 5.4. Inflammatory Bowel Disease

CD and UC are the two major forms of inflammatory bowel disease (IBD). IBD is a chronic condition that affects individuals with a genetic predisposition, resulting in a breakdown in intestinal homeostasis and the development of aberrant inflammatory responses to the intestinal flora [140]. In the peripheral blood, CD4^+^CD25^high^ T cells derived from patients with IBD exhibit sustained suppressive capabilities. Conversely, levels of CD4^+^CD25^high^ and FoxP3^+^ Tregs are increased during remission phases but diminish during active disease. This pattern contrasts with their strong elevation in the peripheral blood of individuals with acute diverticulitis [141]. The diminished production of functional Tregs contributes to the development of intestinal inflammation, ultimately leading to colitis and other related complications [142]. The frequency and suppressive capacity of Tregs were inversely correlated with disease activity in UC patients [143]. The efficacy of transferred Tregs in controlling inflammatory lesions has been demonstrated in multiple models of IBD [144]. In vitro-generated, IL-10 secreting Tr1 cells were effective in preventing inflammation, induced by CD4^+^CD45RB^high^ T cells in severe combined immunodeficiency disease (SCID) mice. Knockout mice lacking the IL-10 gene are a suitable colitis model for evaluating the efficacy of IL-10-producing Tregs in reversing the pathological condition through infusion. This model provided evidence for the crucial role of IL-10-secreting CD4^+^ Tregs, highlighting their immunosuppressive properties and active suppression of pathological immune responses in vivo [29]. Adoptive transfer of CD4^+^CD25^+^ Tregs into c.b-17 SCID immunodeficient mice presenting clinical signs of inflammation was able to ameliorate colitis 2 weeks post-infusion. The first clinical trial involving a single infusion of freshly isolated Tregs derived from peripheral blood mononuclear cells (PBMCs) was initiated in 2012 for patients with active Crohn’s disease. Clinical responses were predominantly observed in patients who received a lower numbers of Tregs, suggesting that a lower level of suppression may be more effective [145]. Together, these findings indicate that CD4^+^CD25^+^CD127^low^FoxP3^+^ Tregs play a distinct causative role in the immunopathogenesis of IBD, and assessment of Treg frequency and function could hold clinical value.

## 6. Therapeutic Approaches to Restore Immune Tolerance

Due to the ability of Tregs to induce stable immune tolerance, and with our increased understanding of their biology, modulating Treg function has emerged as an attractive therapeutic strategy in autoimmune and inflammatory diseases and for enhanced survival of transplants. Several strategies for enhancing Treg function are being actively pursued including low-dose IL-2, IL-2 fusion proteins, and muteins designed to preferentially activate Tregs, CTLA-4 agonists, rapamycin, and adoptive and autologous transfer of Tregs that have shown promising results in preclinical as well as early clinical studies. Engineered Tregs such as chimeric antigen-receptor Tregs, Tregs with recombinant TCRs targeting specific antigens, induced pluripotent stem-cell derived Tregs, and allogenic, off-the-shelf Treg therapies have drawn increased attention. Notwithstanding the promising early clinical trial data from some of these approaches, challenges such as the ease and feasibility of large-scale production of Tregs, and their lineage and functional stability and durability, remain. The success of Treg therapies as a ‘living drug’ to achieve immune tolerance would depend on concerted efforts to overcome these challenges. For a more comprehensive overview of current progress and challenges in Treg therapies, readers are directed to recent reviews by Ho et al., Bittner et al., and Bluestone et al. [146,147,148].

## 7. Future Perspectives

The precise contribution of Tregs to various autoimmune diseases remains unclear, possibly due to the various markers used to define Tregs. Most studies predominantly analyze circulating blood Tregs, which may not represent the real landscape in various solid tissues, since tissue-resident Tregs normally do not recirculate in the blood or lymphatics [149]. Nevertheless, tissue-resident Tregs play a central role in regulating tissue homeostasis. For example, in RA patients there is a notable disparity in the numbers and suppressive capacity of Tregs between peripheral blood and synovial fluid. The synovial fluid tends to have a higher proportion of Tregs with reduced suppressive capabilities compared to the peripheral blood. This emphasizes the significance of examining Tregs in both peripheral blood and peripheral tissues. Further research into the interaction of Tregs with other immune cells and the infiltration of expanded Tregs in disease-relevant tissues will be highly valuable.

**Figure 3:**
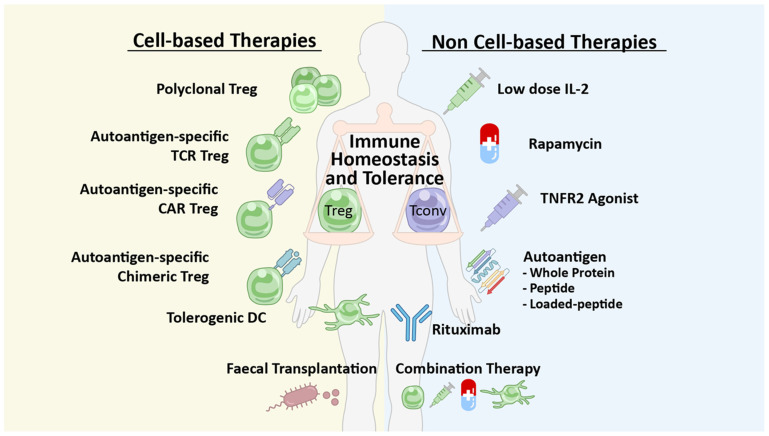
**New therapeutic approaches for autoimmune diseases, encompassing both cell-based and non-cell-based strategies**. Cell-based therapies involve the use of ex vivo expanded polyclonal Tregs or Tregs modified with autoantigen-specific T cell receptors (TCRs), chimeric antigen receptors (CARs), or another chimeric receptor-like peptide-MHC. Another cell-based approach focuses on harnessing the immunomodulatory effects of tolerogenic dendritic cells (DCs). Additionally, fecal transplantation of Treg-promoting bacteria shows promise in restoring immune balance. Non-cell-based therapies encompass biologicals such as low-dose IL-2 therapy, TNFR2 agonist therapy, and an anti-CD20 antibody rituximab. Pharmacological agents such as rapamycin can also enhance Treg proliferation. Furthermore, the administration of autoantigens through various methods enables vaccination against autoimmune responses. Finally, combining these therapies has the potential to yield greater efficacy compared to single interventions. Adapted from [150] “Treg Enhancing Therapies to Treat Autoimmune Diseases” by P. Eggenhuizen, B. H. Ng, and J. D. Ooi, 2020, 21p, 7015, *International Journal of Molecular Sciences.* Abbreviations: CD: cluster differentiation; CAR: chimeric antigen receptor; DC: dendritic cells; IL-2: interleukin 2; Tconv: conventional T cell; TCR: T cell receptor; TNFR2: tumor necrosis factor receptor 2.

A wide variety of cell-based and non-cell-based therapies are focusing on enhancement of the anti-inflammatory or tolerogenic component of the immune system to treat or prevent autoimmune diseases (Figure 3). Non-cell-based therapies such as the mTOR-inhibitor rapamycin and biologicals, including low-dose IL-2, TNF receptor 2 (TNFR2) agonists, or FMS-like tyrosine kinase 3 ligands (Flt3L), are being explored to promote the in vivo induction and expansion of Tregs [150]. Administration of autoantigens could also restore tolerance or be used in vaccines against autoimmunity. Cell-based therapies use expanded Tregs expressing a natural repertoire of polyclonal TCRs, Tr1 cells secreting IL-10, or Tregs, which are ex vivo engineered to target the auto-antigen responsible for exacerbating the autoimmune disease (e.g., a TCR or a chimeric antigen receptor (CAR)). Mature DCs may also be used to expand tolerogenic antigen-specific Tregs [149]. One of the most common and longest-studied approaches is the use of low-dose IL-2 to re-establish a healthy balance between Tregs and Teffs by enhancing the quality and/or quantity of Tregs and subsequently restoring immune tolerance, even in the absence of a pre-existing Treg defect.

In conclusion, Tregs are essential for the maintenance of immune homeostasis and self-tolerance. They suppress the overactivation of immune cells, such as CD4^+^ helper T cells, CD8^+^ T cells, and B cells, preventing excessive inflammation and autoimmune responses. Tregs use various mechanisms, including modulation of dendritic cells, disruption of metabolic pathways, deprivation of IL-2, and secretion of anti-inflammatory cytokines. The classification and identification of Tregs are complex due to the heterogeneity of Treg subsets and functions and various methodologies used for labeling. Dysfunction of Tregs is associated with autoimmune diseases. Expansion or enhancement of their immunosuppression may restore the immune balance and self-tolerance, potentially treating autoimmune disorders.

## Figures and Tables

**Table 1:** Different CD3^+^CD4^+^ Treg subsets and their markers, origin, activation, differentiation factor, and main suppressive mechanism.

Subtype	Markers	Origin	Activation/Differentiation Factor	Main Suppressive Mechanism	Ref.
Naive Treg	CD25^low/−^FoxP3^low/−^CTLA-4^low/−^CD95^−^ CD62L^+^CD45RA^+^CD45RO^low^	Thymus	Antigen-dependent, TCR/CD28, TGF-β	Lack of suppressive activity until they are activated	[17]
Resting Treg	CD25^high^FoxP3^low^Ki-67^−^CD45RA^+^	Thymus/Periphery	TCR stimulation	Not anergic and are able to proliferate upon TCR stimulation	[14]
Activated Treg	CD25^high^ FoxP3^high^ CD95^+^CD45RA^−^ CTLA-4^high^CD45RO^high^	Thymus/Periphery	TCR/CD28, cytokines: IL-2, IL-10, TGF-β	Cell contact, IL-10 and TGF-β, modulation of the metabolism and activation of other immune cells	[14]
Memory Treg	CD25^high^ CTLA-4^high^ CD45RA^low^CD45RO^high^Helios^+^	Thymus/Periphery	Previous antigen stimulation	Lack of suppressive activity until they are reactivated	[14]
Apoptotic Treg	CD95^high^ Bcl-2^low^ Bcl-XL^low^	Thymus/Periphery	Loss of survival signals, increased pro-apoptotic signals, and reduced anti- apoptotic signals	None	[18]
nTreg	CD25^+^CD127^−^CTLA-4^+^GITR^+^Nrp-1^+^ Helios^+^	Thymus	TCR/CD28, affinity-dependent, IL-2	Cell contact	[19,20]
ICOS^+^	ICOS^+^CD25^+^FoxP3^+^	Thymus	TCR/CD28, ICOSL, affinity-dependent, IL-2	IL-10 to suppress dendritic cell function and TGF-β to suppress T cell function	[21]
ICOS^−^	ICOS^−^CD25^+^FoxP3^+^	Thymus	TCR/CD28, affinity-dependent	TGF-β	[21]
HLA-G^+^	HLA-G^+^CD25^−^FoxP3^−^	Thymus	TCR/CD28, affinity-dependent	Cell contact, IL-10 and IL-35, soluble HLA-G5	[22,23,24]
HLA-DR^+^	HLA-DR^+^CD25^high^	Thymus	TCR/CD28, affinity-dependent, IL-2 and TGF-β	Early contact-dependent suppression	[25]
HLA-DR^−^	HLA-DR^−^CD25^high^	Thymus	TCR/CD28 (HLA-DQ, HLA-DP), affinity-dependent, IL-2 or IL-33	Early IL-4 and IL-10 secretion and a late contact-dependent suppression	[25]
iTreg	CD25^+^CTLA-4^+^GITR^+^Nrp-1^−/+?^Helios ^−/+?^ FoxP3^high^LAG-3^+^	Periphery	Antigen-dependent	IL-10, TGF-β	[26,27]
Tr1	CD25^−^CTLA-4^+^GITR^+^FoxP3^low^IL-10^+^	Periphery	Antigen-dependent, IL-10, IFN-α	IL-10	[28,29,30]
Th3	CD25^−/+^CTLA-4^low^GITR^−^FoxP3^?^TGF-β^+^	Periphery	Antigen-dependent, TGF-β	TGF-β	[31,32]
iTr35	CTLA-4^+^ FoxP3^−^ IL-10^−^ TGF-β^−^	Periphery	Antigen-dependent, IL-35	IL-35	[33]
Treg-of-B cell	CD25^+^CTLA-4^+^GITR^+^LAG^+^ICOS^+^OX40^+^ PD1^+^FoxP3^−^IL-10^+^TGF-β^+^	Periphery	Cell-cell contact between B and T cells	Cell contact, IL-10	[34,35]

Abbreviations: CD: cluster differentiation; CTLA-4: cytotoxic T-lymphocyte-associated protein 4; FoxP3: forehead box P3; GITR: glucocorticoid-induced tumor necrosis factor-related receptor; HLA: human leukocyte antigen; ICOS: inducible T-cell costimulatory; IFN: interferon; IL: interleukin; iTreg: inducible Treg; LAG-3; lymphocyte activation gene-3; Nrp1: neuropilin-1; nTregs: natural Treg; PD-1: programmed cell death protein 1; TCR: T cell receptor; TGF-β: transforming growth factor beta; Th: T helper cell.

**Table 2:** Different identification markers for Tregs identified in humans and/or mice, their characteristics, and limitations.

Identification Markers	Human	Mice	Characteristics	Notes/Limitations	Ref.
FoxP3^+^	X	X	FoxP3 expression act as a Treg cell lineage- specific marker and correlates with suppressor activity	Does not control all aspects of Treg biology as thymic CD25^+^Foxp3^−^ Treg precursors are fate committed to the Treg lineage	[39,40]
CD25^+^		X	CD25 expression is crucial for maintaining self-tolerance and dysfunction of this immunoregulation can underly autoimmune diseases	The use of CD25 expression as a marker is limited in human studies	[41]
CD25^high^FoxP3^+^	X		Exhibit strong in vitro regulatory function	No clear boundary between CD25^+^ and CD25^high^ expression	[42]
CD25^+^CD127-	X	X	Express the highest level of FoxP3, effectively suppress the proliferation of CD4^+^CD25^−^ T cells, and no intracellular staining required	CD127 expression alone cannot accurately discriminate Treg cells from activate T cells	[43,44,45]
CD39^high^		X	Exhibit sustained FoxP3 levels, functional suppressive abilities even within an inflammatory environment, no intracellular staining required	-	[46]
CD25^+^GITR^+^	X	X	GITR expression is essential in immunological self-tolerance maintained by Tregs.	-	[47,48]
FoxP3^+^CTLA-4^+^	X	X	CTLA-4 is a key inhibitory molecule that relies on FoxP3 that competes with CD28, leading to a shortened interaction between naïve T cells and APCs, and the ability to inhibit TCR signaling	-	[49,50]
CD25^+^LAG3^+^	X	X	Tregs express LAG-3 upon activation, and Tregs from LAG-3^−^ mice exhibit reduced regulatory activity	-	[51]
CD39^+^CD37^+^	X	X	90% of FoxP3^+^ Tregs are CD39+, CD39 and CD37 generate an immunosuppressed environment (increased adenosine levels)	Not specific for Tregs, expressed on endothelial cells and various immune cells	[37,52]

Abbreviations: APC: antigen presenting cell; CD: cluster differentiation; CTLA-4: cytotoxic T-lymphocyte associated protein 4; FoxP3: forehead box P3; GITR: glucocorticoid-induced tumor necrosis factor-related receptor; LAG-3: lymphocyte activation gene-3; TCR: T cell receptor.

**Table 3:** Different CD3^+^CD4^+^ Treg subsets that have lost (some) suppressive capacity and their markers, differentiation factor, and function.

Subset	Markers	Differentiation Factor	Function	Ref.
Th1-like Tregs	T-bet^high^ CXCR3^+^ FoxP3^+^	T-cell receptor stimulation in the presence of IFN-γ and IL-12	Suppressing Th1-mediated responses, IFN-γ, IL-4 and IL-13	[102]
Th17-like Tregs	FoxP3+CD45RA^−^ ROR-γt^high^ CCR6^+^	T-cell receptor stimulation in the presence of IL-6 and IL-1β	Suppressing Th17-mediated responses, IL-17 secretion	[103,104,107]
Th2-like Tregs	GATA3high ROR-γt-FoxP3^+^	T-cell receptor stimulation in the presence IL-2	Mostly found in tissues. Secreting IL-13, IL-4, and IL-5 chemotaxis to CCL17/22	[108]
Tfh-like (Tfr)	PD-1^high^CXCR5^high^ BCL6^high^	Antigen-dependent, vaccination, infection by influenza, CD28	Suppression of Tfh function and antibody production, prevention of humoral autoimmunity, control of IgE	[109]
exFoxP3 Tregs	CD127^high^ FoxP3^−^	Possible demethylation of FoxP3 triggered by proinflammatory cytokines, such as IL-6/deficiency of IL-2 signaling	Activated-memory T cell phenotype and produced inflammatory cytokines	[110]
Exhauster	PD-1^+^ FoxP3^+^ TIM-3^+^ LAG-3^+^	Facilitated by chronic antigen and inflammation	Reduced suppressive function	[111]

Abbreviations: CD: cluster differentiation; FoxP3: forehead box P3; IFN: interferon; IL: interleukin; LAG-3: lymphocyte activation gene-3; PD-1: programmed cell death protein 1; Tfh: T follicular helper; Th: T helper cell.

## Data Availability

No new data were created or analyzed in this study. Data sharing is not applicable to this article.
